# Expression of SASH1 in Preeclampsia and Its Effects on Human Trophoblast

**DOI:** 10.1155/2020/5058260

**Published:** 2020-10-19

**Authors:** Shisan Liu, Sijia Jiang, Liping Huang, Yanhong Yu

**Affiliations:** Department of Gynecology and Obstetrics, Nanfang Hospital, Southern Medical University, Guangzhou, 510515 Guangdong, China

## Abstract

**Aim:**

To explore the involvement of SASH1 in preeclampsia.

**Methods:**

Expression of SASH1 was determined by qPCR, WB, and immunohistochemistry in the placenta of both normal and preeclamptic pregnancies. The SASH1 gene of human HTR-8/SVneo cells was overexpressed by transfection of pEZ-Lv206-SASH1. After that, the CCK-8 assay, EdU assay, transwell assay, and flow cytometry were used to examine the cell proliferation, migration, invasion, and apoptosis.

**Results:**

Higher expression of SASH1 was detected in placental tissues collected from patients with preeclampsia, compared with those from gestational age-matched control samples. The expression of SASH1 was significantly enhanced by transfection with pEZ-Lv206-SASH1 in HTR-8/SVneo cells. In addition, the HTR-8/SVneo cells transfected with pEZ-Lv206-SASH1 exhibited significantly reduced proliferation, migration, and invasion ability compared to the cells in the empty vector group and normal group. Flow cytometry analysis demonstrated that the apoptosis rate of cells transfected with pEZ-Lv206-SASH1 was significantly higher than that of cells transfected with empty vector and untreated cells.

**Conclusions:**

SASH1 is significantly upregulated in the placenta of preeclampsia, and overexpression of SASH1 can inhibit the proliferation, migration, and invasion, but induce apoptosis of trophoblast cells *in vitro*.

## 1. Introduction

Preeclampsia (PE), a major cause of maternal and perinatal mortality, affects nearly 20% of pregnancy-related mortalities [1, 2]. It is a serious pregnancy disorder, characterized by gestational hypertension and proteinuria in pregnant women, with an incidence of 5% to 7% [3, 4]. The most effective way to cure PE is to terminate pregnancy earlier, therefore leading to an increase of premature births [5]. A leading theory regarding the pathogeny of preeclampsia implicates abnormal early placenta development, trophoblasts invasion, and vessel remodeling [6]. Recently, a lot of researches about the role of genes and proteins in preeclampsia placenta have been introduced.

SASH1 (SAM and SH3 domain-containing 1) is a member of the SLY family of the signal adapter proteins [7]. It has been found in a variety of cancers, such as lung cancer, breast cancer, cervical cancer, ovarian carcinoma, and gastric cancer [8–10], and has been reported as a tumor suppressor promoting cell death and inhibiting the proliferation and invasion of cancer cells [11–14]. Interestingly, RNA sequencing revealed that SASH1 was upregulated in the placenta of preeclampsia patients, implicating its function in preeclampsia [15]. However, the protein expression level and the specific role of SASH1 in preeclampsia have not yet been reported.

In this study, the expression of SASH1 in the placenta of both normal and preeclamptic pregnancies was detected and its correlation to the clinical pathology of preeclamptic was determined by using qRT-PCR, Western blot, and immunohistochemistry analysis. In addition, HTR-8/SVneo cells were transfected with pEZ-Lv206-SASH1, and the biological characteristics of this cell line were clarified to investigate the role of SASH1 in trophoblast. The correlation between SASH1 and trophoblast functions was discussed to provide a new therapeutic target for treating preeclampsia.

## 2. Materials and Methods

### 2.1. Patient Selection

The diagnosis of preeclampsia was based on strict criteria from the American College of Obstetricians and Gynecologists (ACOG), namely, blood pressure higher than 140/90 mmHg after 20 weeks pregnancy and new onset proteinuria more than 0.3 g in 24 h collection. Maternal placenta biopsies were collected from the patients at the time of delivery of preeclampsia (>34 weeks, *n* = 20) and from gestation age-matched normotensive controls (>34, *n* = 20). Patients were excluded if they had a history of chronic hypertension, pregnancy diabetes mellitus, hepatitis, clinical evidence of infection, and autoimmune disease.

This study was approved by Human Research Ethics Committee of Nanfang Hospital, Southern Medical University, China, and written informed consent was obtained from all participants.

### 2.2. Placenta Biopsy Collection

The placental biopsies were collected, from both preeclampsia and normotensive pregnancies, following caesarean sections. It is important to state that only placenta biopsy from women who had not undergone labor during the pregnancy was obtained in order to avoid any effects of labor on the gene expression profiles of the tissue. A central area (approximately 2 cm beside the umbilical cord insertion) of chorionic tissue was dissected, and the amnionic membranes were removed. We then dissected 1 cm sections of placental villi from the four different central areas between the basal and chorionic plates. This standardized location could reduce the bias related to the physiological difference within the same placenta depending on the sampling site. After vigorous washing of the maternal blood with saline, tissues were immediately frozen in liquid nitrogen and stored until use.

### 2.3. qRT-PCR Analysis

RNA was isolated by Trizol reagent (Life, USA) and used to prepare the cDNA following the manufacturer's instructions (Ferments, Canada). qRT-PCR reactions were performed using SYBR green qPCR mix (Takara, China) with Roche Light Cycler 480 (Roche, USA). The primer sequences used for RT-PCR were as follows: SASH1 F: 5′-CGGTCCCAGATCGAAGAGTC-3′ and R: 5′-GTTCTTTCGGAAGTTCTGCCA-3′; *β*-actin F: 5′-GCATCCCCCAAAGTTCACAA-3′ and R: 5′-AGGACTGGGCCATTCTCCTT-3′.

### 2.4. Western Blot

Total protein was lysed in RIPA supplied with 100 *μ*M PMSF (Beyotime, China), and protein concentration was determined using a Bradford Protein Assay Kit (Beyotime, China). Equal amounts of proteins were loaded and analyzed by standard Western blot (10% reducing SDS-PAGE and 0.2 *μ*m PVDF membrane). The membranes with transferred protein were blocked in 5% nonfat dry milk for 2 h at RT and incubated with SASH1 or actin primary antibodies (anti-SASH1, Thermo, USA; *β*-actin, Xingzhi Biotechnology, China) overnight at 4°C, followed by HRP-conjugated IgG second antibody (Xingzhi Biotechnology, China). The immunoreactive protein bands were visualized by ECL kit (Thermo, USA) with Gel Doc (ESCO, Singapore).

### 2.5. Immunohistochemistry

Placental samples were fixed in 10% formaldehyde and embedded in paraffin. Sections (5 *μ*m) were collected onto silane-coated slides. The slides were deparaffinized, rehydrated, treated with 3% hydrogen peroxide for 10 min at RT, and boiled in 0.1 M citrate buffer in a microwave oven for 5 min. Next, the slides were incubated with 5% goat serum to suppress nonspecific binding. Rabbit anti-human SASH1 polyclonal antibody (ab2633692; Thermo, Germany) was used as the primary antibody and incubated at 4°C overnight. Then, the sections were incubated with goat anti-rabbit secondary antibody (Hangzhi Biotechnology, China) for 30 min at 37°C. The sections were detected using DAB as the substrate and were counterstained with hematoxylin for 2 min to reveal the nuclei.

### 2.6. Cell Culture

Human trophoblast cell line HTR-8/SVneo was a kind gift of Dr. Charles H. Graham (Queen's University, Ontario, Canada). Cells were cultured in RPMI-1640 medium (Cellgro, USA) containing 10% FBS (Life, USA) and 100 U/ml PS (Life, USA), at 37°C with 5% CO_2_. Upon reaching 80% confluency, cells were trypsinized with 0.05% trypsin and plated at a density of 6 × 10^5^ cells per well in 6-well plates. Cells were cultured for 24 h followed by transfection with Lipofectamine 2000 (Invitrogen, USA) according to the manufacturer's instructions.

### 2.7. Cell Transfection

An expression plasmid containing SASH1, pEZ-Lv206-SASH1, and an empty vector control plasmid, pEZ-Lv206 (GeneCopoeia, China), were transfected into HTR-8/SVneo cells. Transfections were performed with Lipofectamine 2000 according to the manufacturers' instructions.

The transfection efficiency was determined by detecting the expression of SASH1 using qRT-PCR and WB 36 h after transfection.

### 2.8. Cell Counting Kit-8 (CCK8) Assay

HTR-8/SVneo cells were transfected as described above and cultured for 24 h at 37°C with 5% CO_2_. Cells were trypsinized, resuspended in RPMI-1640 medium supplemented with 10% FBS, counted, and seeded in 96-well plates with a density of 5 × 10^3^ per well. Cells were incubated for 24, 36, 48, 60, and 72 h at 37°C, respectively. After that, 100 *μ*l serum-free RPMI-1640 containing 10% (*v*/*v*) CCK-8 reagents (Dojindo, Japan) was added in each well, and cells were cultured for 1 h at 37°C with 5% CO_2_. Finally, OD_450_ was measured using a microplate reader (MDS, USA).

### 2.9. EdU Assay

HTR-8/SVneo cells were transfected as described above and cultured for 24 h at 37°C with 5% CO_2_. Cells were trypsinized, resuspended in RPMI-1640 medium supplemented with 10% FBS, counted, and seeded in 6-well plates with a density of 5 × 10^4^ per well. After 36 h of incubation at 37°C with 5% CO_2_, 100 *μ*l EdU medium (Ribobio, China) (50 *μ*mol) was added to each well and incubated for 2 h at 37°C with 5% CO_2_. Then, Hoechst 33258 and EdU staining solution were added to each well to mark the living cells (blue) and the proliferating cells (red). Cells stained with blue and red were observed using a fluorescence microscope (Olympus, Japan) and counted using the ImageJ software.

### 2.10. Cell Migration and Invasion Assays

HTR-8/SVneo cells were transfected as described above and cultured for 24 h at 37°C with 5% CO_2_. Cells were trypsinized, resuspended in serum-free RPMI-1640 medium, counted, and 2 × 10^4^ cells in 200 *μ*l were added to chamber inserts (8 *μ*m pore size, Corning, USA). For invasion assays, cells were treated as described, but transwell chamber inserts were precoated with Matrigel (Corning, USA). RPMI-1640 medium supplemented with 10% FBS was added to the lower chambers as a chemoattractant. After 24 h (migrated) or 48 h (invaded) of incubation at 37°C with 5% CO_2_, the cells on the upper surface of the chamber filter were removed by scraping. The migrated/invaded cells on the lower surface of the chamber filter were washed, fixed, and stained with 0.1% crystal violet and visualized by light microscopy (Olympus, Japan). The number of cells migrated through the transwell was measured by counting the migrated cells in 5 random x400 fields for each chamber.

### 2.11. Apoptosis Assay by Flow Cytometry Analysis

HTR-8/SVneo cells were transfected as described above and cultured for 48 h at 37°C with 5% CO_2_. Cells were trypsinized, washed, and resuspended in PBS. Then, 1 × 10^5^ cells were stained using an Annexin V/FITC kit (KeyGen Biotech, China), followed by flow cytometry (BD, USA) detection. The percentage of apoptosis cells was calculated by Annexin V-positivity and PI-positivity.

### 2.12. Statistical Analysis

The SPSS 20.0 statistical software was used for the statistical analysis of the experimental data. One-way ANOVA followed by Tukey's post hoc test (for >2 groups) or a *t*-test (for 2 groups) was used. *P* < 0.05 was considered significant. All data are expressed as the mean ± SE.

## 3. Results

### 3.1. Demographic Data of Study Population

The clinical characteristics of 20 women with preeclampsia (PE group) and 30 women with uncomplicated pregnancies (normal group) are summarized in Table 1. In the two groups, there were no apparent differences in the maternal age. When compared to the normal group, the gestational age was significantly earlier, while the systolic and diastolic blood pressure significantly elevated in the PE groups.

### 3.2. SASH1 Protein Expression in Placentas

**Figure 1:**
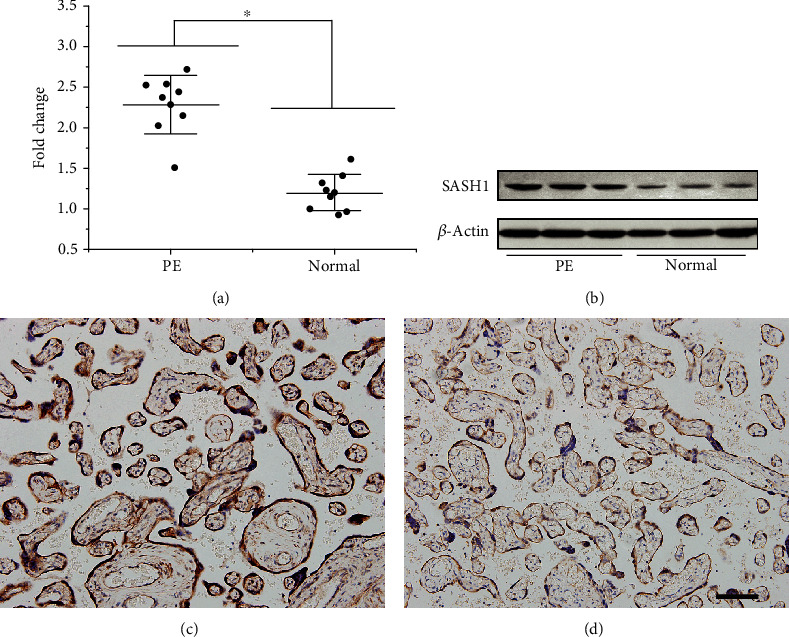
SASH1 expression in human placenta. (a) SASH1 mRNA expression was increased in PE compared to normal placental tissue (^∗^*P* < 0.05, between the PE and normal groups, *n* = 8). (b) WB analysis of SASH1 protein expression in PE and normal placental tissue samples (*n* = 3). (c, d) Immunohistochemical analysis of SASH1 protein expression in human placenta. The signals of SASH1 were localized in the cytoplasm and nucleus of cytotrophoblasts as well as in syncytiotrophoblasts in the placental villi from preeclampsia (c) and normotensive control pregnancies (d). Scale bar = 200 *μ*m; original magnification, ×200.

To verify the expression level of SASH1, placental tissues from women with PE and uncomplicated pregnancies were collected. There was a significant increase in SASH1 expression in both mRNA (Figure 1(a), *n* = 8) and protein (Figure 1(b), *n* = 3) in PE compared to normal samples. The location and the level of SASH1 protein expression were determined by immunohistochemistry. As shown in Figures 1(c) and 1(d), higher SASH1 expression was observed in the cytoplasm and nucleus of cytotrophoblasts as well as in syncytiotrophoblasts in the placental villi from preeclampsia.

### 3.3. Expression of SASH1 in HTR-8/SVneo Cells after Transfection

**Figure 2:**
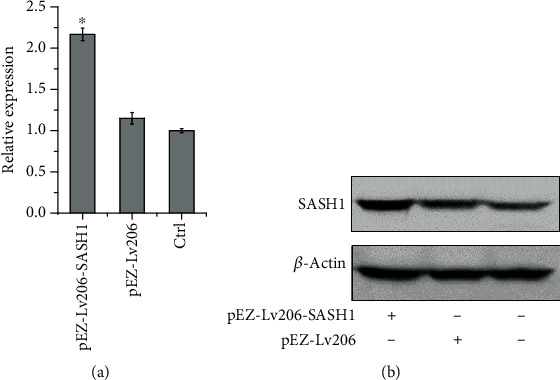
Expression of SASH1 in HTR-8/SVneo cells 36 h after transfection. mRNA and protein expressions of SASH1 in pEZ-Lv206-SASH1, pEZ-Lv206-transfected cells, and untreated cells determined by qRT-PCR (a) and WB (b), respectively. ^∗^*P* < 0.05, compared to the other two groups.

The transfection efficiency was determined by detecting the expression of SASH1 using qRT-PCR and WB. As shown by Figure 2(a), qRT-PCR data revealed pronounced efficacy after transfection. The SASH1 gene was significantly upregulated in the pEZ-Lv206-SASH1 group compared to both untreated and pEZ-Lv206 groups. Moreover, the expression of SASH1 was assessed by WB (Figure 2(b)). The expression of SASH1 was significantly enhanced by transfection with pEZ-Lv206-SASH1, whereas no change was observed between the pEZ-Lv206 and control groups.

### 3.4. Effect of SASH1 Overexpression on HTR-8/SVneo Cell Proliferation

**Figure 3:**
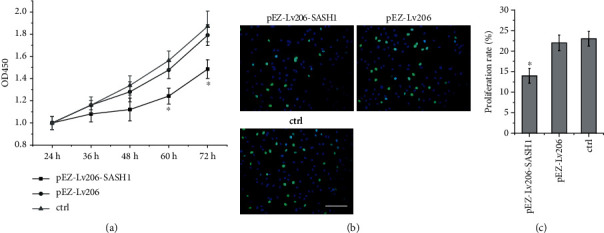
Results of SASH1 gene overexpression on proliferation of HTR-8/SVneo cells. (a) CCK8 assay (^∗^*P* < 0.05, compared to both other two groups at 60 and 72 h); (b) EdU assay; the proliferation cells were stained as green; (c) proliferation rate of cells was calculated (^∗^*P* < 0.05, compared to other groups). Scale bar = 200 *μ*m.

HTR-8/SVneo cells were transfected with pEZ-Lv206-SASH1 and pEZ-Lv206 for 24 h, followed by the detection of cell proliferation using CCK-8 and EdU assay. These data revealed that there was no significant difference between the untreated cells and pEZ-Lv206-transfected cells (Figures 3(a)–3(c)). However, the proliferation of cells transfected with pEZ-Lv206-SASH1 was significantly inhibited at 60 and 72 h, compared to proliferation of both the pEZ-Lv206-transfected and untreated cells (Figure 3(a)).

### 3.5. Effect of SASH1 Overexpression on HTR-8/SVneo Cell Migration and Invasion

**Figure 4:**
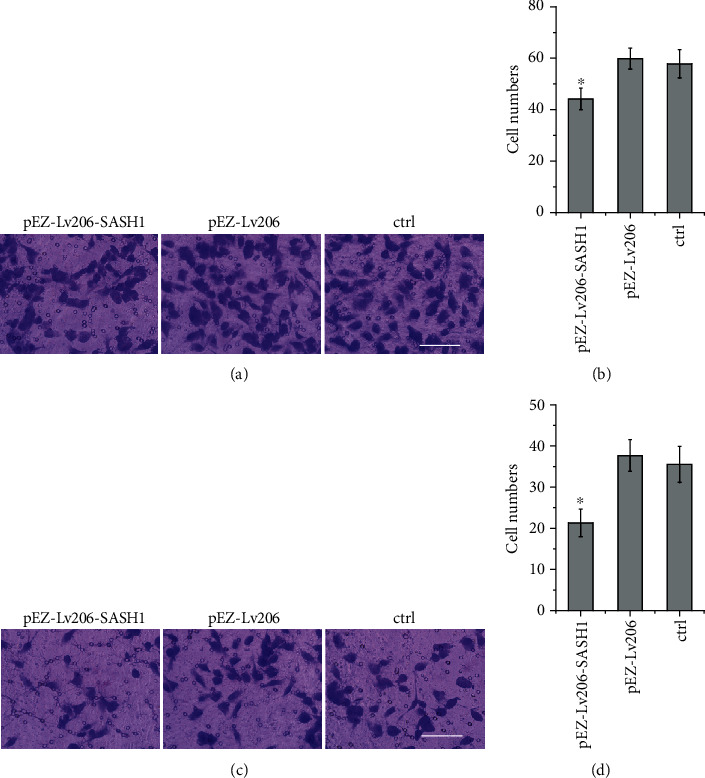
Results of SASH1 gene overexpression on migration and invasion of HTR-8/SVneo cells. (a) Cell migration assay was performed; (b) cells migrated to the down chamber was calculated (^∗^*P* < 0.05, compared to other groups); (c) cell invasion assay was performed; (d) cell invasion to the down chamber was calculated (^∗^*P* < 0.05, compared to other groups). Scale bar = 200 *μ*m.

The migration abilities of SASH1 overexpression-treated HTR-8/SVneo cells were evaluated by transwell assay. HTR-8/SVneo cells were transfected and cultured for 24 h, then seeded in the transwell chamber. There were fewer cells transfected with pEZ-Lv206-SASH1 migrated through the transwell membrane than cells transfected with the pEZ-Lv206 and control groups (Figures 4(a) and 4(b)). These results indicated that overexpression of SASH1 gene could significantly inhibit cell migration.

Like the results of migration assay, overexpression of SASH1 significantly reduced the cell numbers invaded through the chamber membrane, indicating that overexpression of SASH1 could effectively suppress invasion of HTR-8/SVneo cells (Figures 4(c) and 4(d)).

### 3.6. Effect of SASH1 Overexpression on HTR-8/SVneo Cell Apoptosis

**Figure 5:**
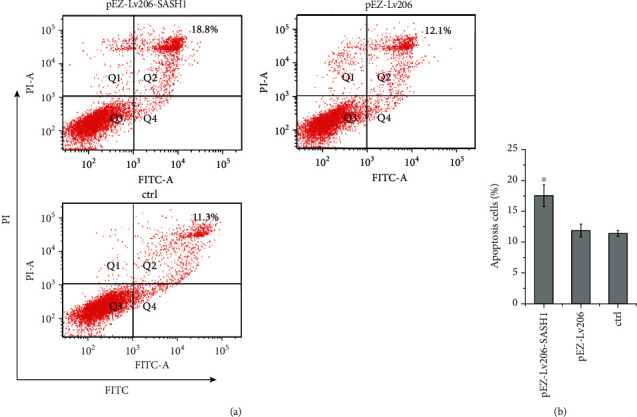
Results of SASH1 gene overexpression on apoptosis of HTR-8/SVneo cells. (a) Apoptosis analysis by flow cytometry; (b) apoptosis rate of cells was calculated (^∗^*P* < 0.05, compared to other groups).

From our present investigation, flow cytometry analysis demonstrated that the apoptosis rate of cells transfected with pEZ-Lv206-SASH1 was 18.8%, significantly higher than that of cells transfected with pEZ-Lv206 (11.8%) and untreated cells (12.3%) (Figure 5). These results revealed that SASH1 overexpression could significantly increase apoptosis of HTR-8/SVneo cells.

## 4. Discussion

SASH1 mRNA has been found upregulated in the placenta of preeclampsia by RNA sequencing compared to that of healthy pregnancy [15]. The results of this study were consistent with this RNA sequencing data that the expression of SASH1 protein was significantly higher in the placenta of preeclampsia than that of the normotensive control pregnancies. SASH1 protein was expressed in the cytoplasm and nucleus of the placental trophoblast cells, suggesting that SASH1 gene might play a role in abnormal placental development in preeclampsia patients. But it is uncertain whether the upregulation of SASH1 in the placenta is the cause of trophoblast insufficient invasion resulting in preeclampsia or a consequence of changes in the uteroplacental microenvironment.

A leading theory states that improper trophoblast invasion results in insufficient vessel remodeling, which causes preeclampsia [16]. The characteristics of trophoblast cells are similar to those of tumor cells, while upregulated SASH1 could enhance cell death and inhibit the proliferation and invasion of tumor cells [11, 17–19]. Therefore, it is reasonable to hypothesize that SASH1 gene might play a part in abnormal placental development in preeclampsia and the upregulation of SASH1 protein might be related to inadequate trophoblast cell invasion in the placental tissues of preeclampsia patients.

To investigate this hypothesis, we overexpressed the SASH1 gene of human HTR-8/SVneo cells and detect their functions, including proliferation, migration, invasion, and apoptosis. HTR-8/SVneo is a trophoblast cell line established by Graham et al. [20] and displays trophoblast progenitor cell-like characteristics and is often used to model the invasion of trophoblast [21].

The expression of SASH1 was significantly enhanced by transfection with pEZ-Lv206-SASH1 in HTR-8/SVneo cells as determined by qPCR and WB (Figure 2). CCK8 and EdU assays showed that overexpression of SASH1 could inhibit the proliferation of HTR-8/SVneo cells (Figure 3). A previous study demonstrated that silencing SASH1 can increase the expression of cyclin D1 (CCND1) and cyclin D3 (CCND3) in human aortic endothelial cells (HAECs). Both CCND1 and CCND3 cyclins are part of the Cyclin-dependent protein kinase complex and are required for G1/S phase transition [22]. Therefore, the inhibition of HTR-8/SVneo cell proliferation by SASH1 may be due to the inhibition of CCND1 and CCND3 expression, by which the cells were arrested in G1 phase.

In addition to inhibiting cell proliferation, overexpression of SASH1 could inhibit cell migration as shown by transwell assay (Figures 4(a) and 4(b)). The first step of cell migration is the formation of lamellipodia [23]. SASH1 was enriched in lamellipodia [12]. Lamellipodia contain abundant F-actin, while F-actin binding protein (cortactin) plays an important role in cell migration and adhesion [24]. Some cortactin binding chaperones are rich in proline sequences, which can interact with the SH3 region of cortactin [25]. There are proline-rich sequences near the C-terminal of SASH1, suggesting the interaction between SASH1 and cortactin. Therefore, SASH1 may regulate trophoblast cell migration by binding with cortactin.

In tumor cells, SASH1 has a significant effect on cell invasion [11–14]. Similarly, this study found that SASH1 can inhibit the invasion of trophoblasts. The key of tumor cells metastasis is the degradation of extracellular matrix and basement membrane [13]. Matrix metalloproteinase (MMP) can degrade laminin and reduce cell adhesion. MMP-2/9 is a major member of matrix metalloproteinase family, which is involved in the degradation of extracellular matrix. It can degrade almost all extracellular matrixes [13] and promote the invasion and metastasis of tumor cells. SASH1 gene may inhibit the invasion and metastasis of tumor cells by inhibiting the expression of MMP-2/9 in A549 cells [24]. Studies have shown that trophoblasts can express MMP2/9, which can degrade the basement membrane and facilitate cell invasion. The synthesis and activation of MMP2/9 play an important role in trophoblast invasion [8, 10]. Therefore, SASH1 may affect the invasion of trophoblast by affecting the expression of MMP2/9.

Apoptosis also occurs in the extravillous trophoblast in preeclampsia in association with abnormal invasion. Little apoptosis of trophoblast was seen in healthy pregnancies, while apoptosis of trophoblasts that invade the uterus was widespread in preeclampsia [26, 27]. Flow cytometry analysis exhibited that SASH1 overexpression could significantly increase trophoblast cell apoptosis, suggesting that SASH1 may induce preeclampsia by promoting apoptosis of trophoblast. Previous studies in HeLa cells showed that SASH1 could be cleaved by caspase-3 during apoptosis, and the C-terminal fragment was transferred from the cytoplasm to the nucleus with the help of NF-*κ*B [14]. The effect of SASH1 was dependent on NF-*κ*B, as confirmed by DHMEQ, an NF-*κ*B inhibitor [14]. However, whether the mechanism of apoptosis induced by SASH1 depends on NF-*κ*B remains to be further studied.

In this research, the results demonstrated that SASH1 gene could inhibit the proliferation, migration, and invasion of trophoblast HTR-8/SVneo cells and promote their apoptosis. Therefore, it is possible that elevated expression of SASH1 during the development of placenta could potentially affect trophoblast migration and invasion, leading to shallow trophoblast cell invasion and poor arteriole remodeling in the placental tissues of preeclampsia patients. Nonetheless, the only cell line used in this study may impact the credibility and generalizability. Further research is needed to confirm the overexpression effects on other trophoblast cell lines and primary trophoblast and to distinguish in which signaling pathway SASH1 affects trophoblast. The study not only can be used for early diagnosis, treatment, and prognosis judgment but also can help to develop new treatment methods for preeclampsia.

## 5. Conclusions

The present study reveals that SASH1 is significantly upregulated in the placenta of preeclampsia and overexpression of SASH1 can significantly suppress the proliferation, migration, and invasion but induce apoptosis of trophoblast cells in vitro. These results suggest that SASH1 might provide a new therapeutic target for treating preeclampsia.

## Figures and Tables

**Table 1:** Comparison of clinical indexes between the normal pregnant group and the preeclampsia group ($\bar{x}\pm s$).

Group	*n*	Year	Gestational age (week)	Diastolic blood pressure (mmHg)	Systolic blood pressure (mmHg)
Normal	30	29.13 ± 3.72	38.73 ± 0.74^∗^	78.43 ± 6.72	118.47 ± 6.68^∗^
Preeclampsia	20	29.00 ± 2.80	36.50 ± 1.24	83.25 ± 10.49	159.20 ± 17.86
*t*		0.137	7.263	1.983	9.753
*P*		0.119	0.0001	0.053	0.0001

^∗^ *P* < 0.05 vs. preeclampsia.

## Data Availability

The data used to support the findings of this study are included within the article.
